# Risk factors for a serious adverse outcome in neonates: a retrospective cohort study of vaginal births

**DOI:** 10.1111/1471-0528.17531

**Published:** 2023-05-08

**Authors:** Sita Jindal, Philip J. Steer, Makrina Savvidou, Tim Draycott, Mary Dixon‐Woods, Angela Wood, Lois G. Kim

**Affiliations:** ^1^ Academic Department of Obstetrics and Gynaecology Imperial College London, Chelsea and Westminster Hospital London UK; ^2^ The Royal College of Obstetricians and Gynaecologists London UK; ^3^ Department of Women's Health North Bristol NHS Trust Westbury on Trym UK; ^4^ Department of Public Health and Primary Care University of Cambridge, Strangeways Research Laboratory Cambridge UK; ^5^ Department of Public Health and Primary Care / Cardiovascular Epidemiology Unit, Victor Phillip Dahdaleh Heart and Lung Research Institute University of Cambridge Cambridge UK; ^6^ Health Data Research UK Cambridge Wellcome Genome Campus and University of Cambridge Cambridge UK

**Keywords:** fetal deterioration, intrapartum fetal monitoring, labour outcome, meconium, pyrexia, risk factors

## Abstract

**Objective:**

To investigate the hypothesis that risk factors in addition to an abnormal fetal heart rate pattern (aFHRp) are independently associated with adverse neonatal outcomes of labour.

**Design:**

Observational prospective cohort study.

**Setting:**

17 UK maternity units.

**Sample:**

585 291 pregnancies between 1988 and 2000 inclusive.

**Methods:**

Adjusted odds ratios (OR) with 95% confidence intervals (95% CI) were estimated from multivariable logistic regression.

**Main outcome measures:**

Adverse neonatal outcome at term (5‐minute Apgar score <7, and a composite measure comprising 5‐minute Apgar score <7, resuscitation by intubation and/or perinatal death).

**Results:**

Analysis was based on 302 137 vaginal births at 37–42 weeks inclusive. We found a higher odds of Apgar score at 5 minutes <7 with suspected fetal growth restriction (OR 1.34, 95% CI 1.16–1.53), induction of labour (OR 1.41, 95% CI 1.25–1.58), nulliparity (OR 1.48, 95% CI 1.34–1.63), booking body mass index ≥30 (OR 1.18, 95% CI 1.02–1.37), maternal age <25 (OR 1.23, 95% CI 1.10–1.39), black ethnicity (OR 1.21, 95% CI 1.03–1.43), early‐term birth at 37–38 weeks (OR 1.13, 95% CI 1.02–1.25), late‐term birth at 41–42 weeks (OR 1.14, 95% CI 1.01–1.28), use of oxytocin (OR 1.27, 95% CI 1.14–1.41), maternal pyrexia (OR 1.87, 95% CI 1.46–2.40), aFHRp and presence of meconium (aFHRp without meconium: OR 2.40, 95% CI 2.15–2.69; meconium without aFHRp: OR 2.20, 195% CI.94–2.49; both aFHRp and meconium: OR 4.26, 95% CI 3.74–4.87). The results were similar when the composite adverse outcome was considered.

**Conclusions:**

A range of risk factors, including suspicion of fetal growth restriction, maternal pyrexia and presence of meconium, are implicated in poor birth outcomes in addition to aFHRp. Interpretation of the fetal heart rate pattern alone is insufficient as a basis for decisions about escalation and intervention.

## 1 INTRODUCTION

Suboptimal monitoring and response to fetal deterioration during labour have been repeatedly identified as being among the most common contributory factors to poor outcomes at birth, with around 70% of successful maternity litigation claims associated with deficiencies in intrapartum monitoring and care.^1^, ^2^ In the UK, around 120–130 babies die annually during labour (intrapartum stillbirth; 20/100 000), 145–156 die in the first week of life (early neonatal death; 25/100 000) and 850–860 survive with severe brain injury (140/100 000),^3^ notwithstanding some debate on the definition of such injuries.^4^ Many of these poor outcomes are associated with substandard intrapartum care,^3^ including preventable errors in fetal surveillance and response, which are present in 72–76% of cases.^5^, ^6^, ^7^

Despite its limited benefits, cardiotocography (CTG) has become the standard for intrapartum fetal monitoring in high‐income countries, especially in managing high‐risk pregnancies.^8^, ^9^, ^10^, ^11^ However, the 2019 Each Baby Counts report^6^ found that abnormality of the fetal heart rate pattern (FHRp) is only one risk factor relevant to outcomes. Moreover, abnormal fetal heart rate pattern (aFHRp) is common, and while a normal pattern is a reliable predictor of a good outcome, an abnormal pattern has only a modest correlation with a poor outcome given high false‐positive rates.^12^ The 2015 Each Baby Counts report identified an average of six risk factors per labour associated with poor outcomes: suspected fetal growth restriction (FGR, in our study based on there being >3 antenatal ultrasound scans, the number of which correlated highly with birthweight <5th percentile, quadratic *R* ^2^ 0.979), previous caesarean section, intrapartum vaginal bleeding, prolonged labour, meconium‐stained amniotic fluid and suspected infection (usually recognised as maternal pyrexia).^13^ These findings are consistent with other studies showing an association between poor outcomes of labour and a wider range of risk factors other than FHRp alone.^3^, ^14^, ^15^ However, few studies to date have offered clear estimates of the strength of association.

The National Institute for Health and Clinical Excellence (NICE) updated 2017 guideline on intrapartum care for healthy women and babies highlighted that clinical decision‐making should include ‘maternal and fetal risk factors' in addition to fetal heart rate abnormalities.^16^ However, the guidance does not specify the ‘risk factors' other than ‘vaginal bleeding’ and ‘significant meconium’, does not quantify them, and does not discuss the significance of combinations of risk factors.

We therefore investigated the hypothesis that risk factors in addition to an aFHRp are independently associated with a 5‐minute Apgar score <7, and a composite adverse neonatal outcome of labour, defined as 5‐minute Apgar score <7, resuscitation by intubation and/or perinatal death.

## 2 METHODS

### 2.1 Data

We used data collected from maternity units in the former North West Thames Regional Health Authority (RHA) area^16^ that used the St Mary's Maternity Information System (SMMIS), a computerised records system for every pregnancy booked at these units from 1988 to 2000 inclusive (there were 17 units initially but 2 × 2 units subsequently merged, so there were 15 in 2000). Data were collected prospectively for all pregnancies (*n* = 585 291) booked at the participating units from the first antenatal visit up to 28 days postpartum by trained clerks or midwives. The dataset includes demographic data and information about pregnancy, labour, birth and pregnancy outcome. The data entry system used online computer validation using defined ranges and cross‐checks to minimise data entry errors, computer prompting and standard definitions for clinical measurements recorded onto an electronic data storage system. The data collection methods have been extensively described previously and the data have been used in many published studies.^12^, ^16^, ^17^, ^18^

We excluded any labours without a recorded gestational age at birth (the majority lost to follow‐up because they moved away from the region) and pre‐labour caesarean sections. We also excluded multiple pregnancies, breech/non‐vertex presentations and preterm/post‐term births (<37 and >42 completed weeks, respectively). The flow chart of exclusions is shown in Figure S1.

There was no patient or public involvement (PPI) in the design and planning of this study: it is a retrospective, statistically driven analysis of a previously collected dataset where opportunities for meaningful patient involvement were limited.

### 2.2 Outcomes

We investigated two outcomes. First, we used 5‐minute Apgar score <7 as a useful proxy for an adverse outcome, given that infants born with this indicator have an increased risk of hypoxic brain injury,^4^ cerebral palsy^19^ and neonatal death.^20^ Around 3% of infants with 5‐minute Apgar score <7 will develop cerebral palsy, with hazard ratios of between 15.4 and 227.7 times the reference rate.^20^ This measure also occurs frequently enough to allow for reliable statistical analysis.^20^, ^21^, ^22^

Secondly, we used a composite outcome comprising any one of (i) 5‐minute Apgar score <7, (ii) neonatal resuscitation by intubation and positive pressure ventilation (with or without cardiac massage) and (iii) perinatal death (intrapartum and early neonatal death), a rare but important outcome. We included perinatal death as well as the Apgar score in the composite outcome because it is the most important outcome to avoid, and we included intubation as a major intervention requiring expert input, particularly relevant in relation to meconium‐stained amniotic fluid, and because it can itself modify the Apgar score.

We considered a range of well‐established demographic, antenatal and intrapartum risk factors^3^, ^4^, ^5^, ^6^, ^15^ for which data were available in our dataset (Figure S2). Where judgement was involved, the criteria for entry was that the clinicians involved in the labour thought the risk factor was significant. For example, there was no attempt to determine whether meconium staining of the amniotic fluid was thick or thin, old or new, simply whether the birth attendants thought it was clinically significant to record it. The intrapartum heart rate was recorded as whether cardiotocography (CTG) patterns were normal, abnormal or ‘not done’, i.e. intermittent auscultation only was performed. The policy during the study period was for intermittent auscultation in labours with no risk factors but for cardiotocography to be done if risk factors developed (including abnormal rates on auscultation). ‘Not done’ is therefore interpreted as ‘heart rate not abnormal’. The numbers were 54 395/302 137 (18%) with abnormal CTG, 227 290 (75%) with normal CTG and 20 452 (7%) not done.

### 2.3 Statistical methods

We used multivariable logistic regression to estimate odds ratios for the association between risk factors and the two outcomes of interest. As the outcomes of interest are rare, we interpret results as risks, for readability. We excluded records with missing data (risk factors or outcomes) from the primary analysis. If any component was missing in the composite outcome, the outcome was classified as missing.

Caesarean section is designed to prevent adverse outcomes and therefore alters the association of risk factors with outcome. Given substantial differences between those undergoing emergency caesarean and those giving birth vaginally or through planned caesarean, we conducted our analysis on labours that did not result in emergency caesarean section, i.e. those that were delivered vaginally.

In sensitivity analyses, we addressed the missing data using multiple imputation. Missing values were generated for risk factors and outcome components using chained equations to create 25 imputed datasets (based on the overall proportion with missingness) with 10 burn‐ins and augmentation to facilitate fitting. Binary variables (parity, previous caesarean section, suspected FGR, induction of labour, antepartum haemorrhage, epidural use, oxytocin use, maternal pyrexia, aFHRp, presence of meconium and the three components of the outcome: 5‐minute Apgar score <7, resuscitation and perinatal death) were imputed using logistic regression. Ethnicity was imputed using multinomial logit and gestational age category using ordered logit. To account for skewness, booking body mass index (BMI) was imputed using predictive mean matching for underlying original values of BMI, then categorised after imputation. Previous caesarean section was imputed conditionally for those imputed as non‐nulliparous (i.e. nulliparity and previous caesarean section were treated as mutually exclusive). Induction of labour and use of oxytocin were each excluded from imputation of the other, with the aim of avoiding implausible imputed combinations of these factors. Interactions between each outcome component and meconium/aFHRp were included in the respective imputation models to reflect the inclusion of the aFHRp–meconium interaction in the analytical model. Missing outcome components were also imputed and included in post‐imputation analysis, as the combination of these into a composite outcome confers some further gains. Results from the 25 imputations were combined using Rubin's rules to provide pooled estimates.

Results are expressed as odds ratio (OR) with 95% confidence intervals (CI). Analysis was carried out using STATA version 16.1.

## 3 RESULTS

### 3.1 Study population and characteristics

Of the 585 291 pregnancies in the dataset, 428 742 term labours met the eligibility criteria (Figure S1). Initial analysis showed that birth by emergency caesarean section was a major effect modifier and was itself associated with significantly different maternal and labour risk factors (Table 1). We therefore elected to exclude 30 758 such births and study only the association of risk factors with outcome in vaginal births. A further 95 847 labours with missing data were then excluded from initial analysis. This left a total of 302 137 labours resulting in vaginal birth to be included in the primary analysis (Table 1, Figure S1).

**TABLE 1:** Baseline characteristics and summary of risk factors for complete case analysis.

Characteristics	All (*n* = 324 644)	Vaginal birth (*n* = 302 137) (93.1%)	Emergency CS delivery (*n* = 22 507) (6.9%)	*P*‐value (χ^2^)
Antenatal variables				
Suspected fetal growth restriction				
No	296 857 (91.4%)	277 040 (91.7%)	19 817 (88.0%)	<0.001
Yes	27 787 (8.6%)	25 097 (8.3%)	2690 (12.0%)	
Previous CS				
No	311 308 (95.9%)	291 912 (96.6%)	19 396 (86.2%)	<0.001
Yes	13 336 (4.1%)	10 225 (3.4%)	3111 (13.8%)	
Nulliparity				
No	177 334 (54.6%)	170 952 (56.6%)	6382 (28.4%)	<0.001
Yes	147 310 (45.4%)	131 185 (43.4%)	16 125 (71.6%)	
Antepartum haemorrhage				
No	317 802 (97.9%)	295 942 (98.0%)	21 860 (97.1%)	<0.001
Yes	6842 (2.1%)	6195 (2.0%)	647 (2.9%)	
Booking body mass index				
Normal, 18–24 kg/m^2^	203 824 (62.8%)	192 056 (63.6%)	11 768 (52.3%)	<0.001
Underweight, <18 kg/m^2^	10 536 (3.3%)	10 071 (3.3%)	465 (2.1%)	
Overweight, 25–29 kg/m^2^	79 705 (24.5%)	72 936 (24.1%)	6769 (30.1%)	
Obese, ≥30 kg/m^2^	30 579 (9.4%)	27 074 (9.0%)	3505 (15.5%)	
Maternal age				
<25	76 515 (23.6%)	72 224 (23.9%)	4292 (19.1%)	<0.001
25–27	63 277 (19.5%)	59 088 (19.5%)	4189 (18.6%)	
28–31	94 261 (29.0%)	87 684 (29.0%)	6577 (29.2%)	
32–34	49 882 (15.4%)	46 100 (15.3%)	3782 (16.8%)	
≥35	40 708 (12.5%)	37 041 (12.3%)	3667 (16.3%)	
Maternal ethnicity				
White	240 347 (74.0%)	225 681 (74.7%)	14 666 (65.2%)	<0.001
Black	18 557 (5.7%)	16 435 (5.4%)	2122 (9.4%)	
Other	65 740 (20.3%)	60 021 (19.9%)	5719 (25.4%)	
Gestational age at delivery				
37–38 weeks	56 088 (17.3%)	52 945 (17.5%)	3143 (14.0%)	<0.001
39–40 weeks	186 832 (57.6%)	176 047 (58.3%)	10 785 (47.9%)	
41–42 weeks	81 724 (25.2%)	73 145 (24.2%)	8579 (38.1%)	
Intrapartum variables				
Induction of labour				
No	264 434 (81.5%)	249 934 (82.7%)	14 500 (64.4%)	<0.001
Yes	60 210 (18.6%)	52 203 (17.3%)	8007 (35.6%)	
Epidural use				
No	246 392 (75.9%)	237 640 (78.6%)	8752 (38.9%)	<0.001
Yes	78 252 (24.1%)	64 497 (21.4%)	13 755 (61.1%)	
Oxytocin use				
No	250 824 (77.3%)	236 198 (78.2%)	14 626 (65.0%)	<0.001
Yes	73 820 (22.7%)	65 939 (21.8%)	7881 (35.0%)	
Maternal pyrexia				
No	320 249 (98.7%)	298 963 (98.9%)	21 286 (94.6%)	<0.001
Yes	4395 (1.4%)	3174 (1.1%)	1221 (5.4%)	
Abnormal FHR				
No	256 359 (79.0%)	247 742 (82.0%)	8617 (38.3%)	<0.001
Yes	68 285 (21.0%)	54 395 (18.0%)	13 890 (61.7%)	
Meconium‐stained amniotic fluid				
No	267 724 (82.5%)	252 816 (83.7%)	14 908 (66.2%)	<0.001
Yes	56 920 (17.5%)	49 321 (16.3%)	7599 (33.8%)	

CS, caesarean section; FHR, fetal heart rate.

Most risk factors had low (<0.5%) missingness, with the exception of maternal ethnicity (2.8%), BMI (20.4%), suspected FGR (1.2%) and antepartum haemorrhage (0.6%). Of those without emergency caesarean, 54 395 (18%) of fetuses had an aFHRp during labour and 49 321 (16%) had meconium‐stained amniotic fluid. Among the outcome variables, missingness was <0.5%. A total of 5521 vaginal births (1.8%) had a serious adverse outcome, including 2147 (0.7%) with a 5‐minute Apgar score <7, 4089 (1.4%) resuscitations by intubation and 105 (0.03%) perinatal deaths (some labours had combinations of these events).

### 3.2 Risk factor associations with 5‐minute Apgar score <7

**FIGURE 1:**
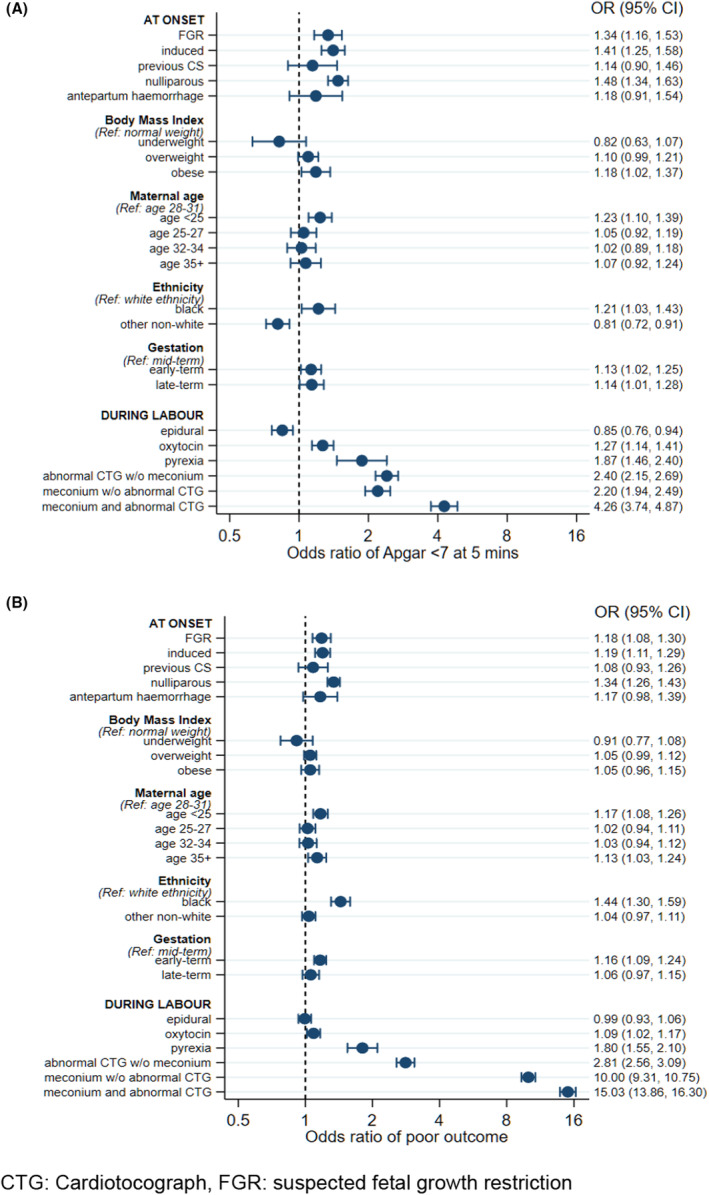
Adjusted odds ratios in women not undergoing emergency caesarean section (complete data, *n* = 302 137) for (A) 5‐minute Apgar <7 and (B) composite poor outcome. Variable definitions are listed in Figure S2.

Suspected FGR, induction of labour and nulliparity were all independently associated with a 34–50% higher risk of the 5‐minute Apgar <7 (Figure 1), all *P* ≤ 0.001, after adjustment for other risk factors. Being obese was associated with an 18% higher risk (OR 1.18, 95% CI 1.02–1.37) and maternal age was also associated with risk of 5‐minute Apgar <7, with those aged <25 (OR 1.23, 95% CI 1.10–1.39) having a higher risk than those aged 28–31. Black ethnicity was associated with a 21% higher risk (OR 1.21, 95% CI 1.03–1.43), early‐term births with a 14% higher risk (OR 1.14, 95% CI 1.01–1.28) and late‐term births with a 13% higher risk (OR 1.13, 95% CI 1.02–1.25).

Use of epidural analgesia was associated with a lower risk of a 5‐minute Apgar score <7 (OR 0.85, 95% CI 0.76–0.94) and this is likely to reflect unmeasured markers of healthy labour progression in those deemed suitable to receive an epidural (for example, pyrexia is a contraindication). Pyrexia during labour was associated with an 87% higher risk (OR 1.87, 95% CI 1.46–2.40) and use of oxytocin was associated with a 27% increased risk (OR 1.27, 95% CI 1.14–1.41) of a 5‐minute Apgar score <7.

Abnormal fetal heart rate pattern without any evidence of meconium‐stained amniotic fluid was associated with a two‐fold higher risk of a 5‐minute Apgar score <7 (OR 2.40, 95% CI 2.15–2.69) and meconium in the absence of aFHRp was similarly associated with a two‐fold higher risk (OR 2.20, 95% CI 1.94–2.49). Vaginal births with both meconium and aFHRp had a four‐fold higher risk (OR 4.26, 95% CI 3.74–4.87) compared with vaginal births with neither meconium nor aFHRp.

There was no evidence to suggest an association between a 5‐minute Apgar score <7 and previous caesarean section or antepartum haemorrhage.

### 3.3 Risk factor associations with composite outcome

The associations of risk factors with the composite outcome were broadly similar to those for 5‐minute Apgar score <7 (Figure 1B). Suspected FGR, induction of labour and nulliparity were all independently associated with an 18–34% increase in risk of the composite outcome (Figure 1), all *P* ≤ 0.001 after adjustment for other risk factors. Maternal age was also associated with risk of composite outcome, with those aged <25 (OR 1.17, 95% CI 1.08–1.26) or ≥35 (OR 1.13, 95% CI 1.03–1.24) having a higher risk than those aged 28–31. Black ethnicity was associated with a 44% higher risk (OR 1.44, 95% CI 1.30–1.59) but there was no evidence of an association between the BMI, previous caesarean section, antepartum haemorrhage or epidural use and composite outcome.

Use of oxytocin during labour was associated with a 9% increase (OR 1.09, 95% CI 1.02–1.17) and pyrexia with an 80% higher risk (OR 1.80, 95% CI 1.55–2.10). An aFHRp without any evidence of meconium in the amniotic fluid was associated with almost a three‐fold higher risk for the composite poor outcome (OR 2.81, 95% CI 2.56–3.09). Meconium on its own, without an aFHRp, was associated with a 10‐fold higher risk (OR 10.0, 95% CI 9.31–10.7). When both meconium and aFHRp were present, vaginal births had a 15‐fold higher risk (OR 15.0, 95% CI 13.9–16.3) than did vaginal births that had neither meconium nor aFHRp.

### 3.4 Sensitivity analyses

**FIGURE 2:**
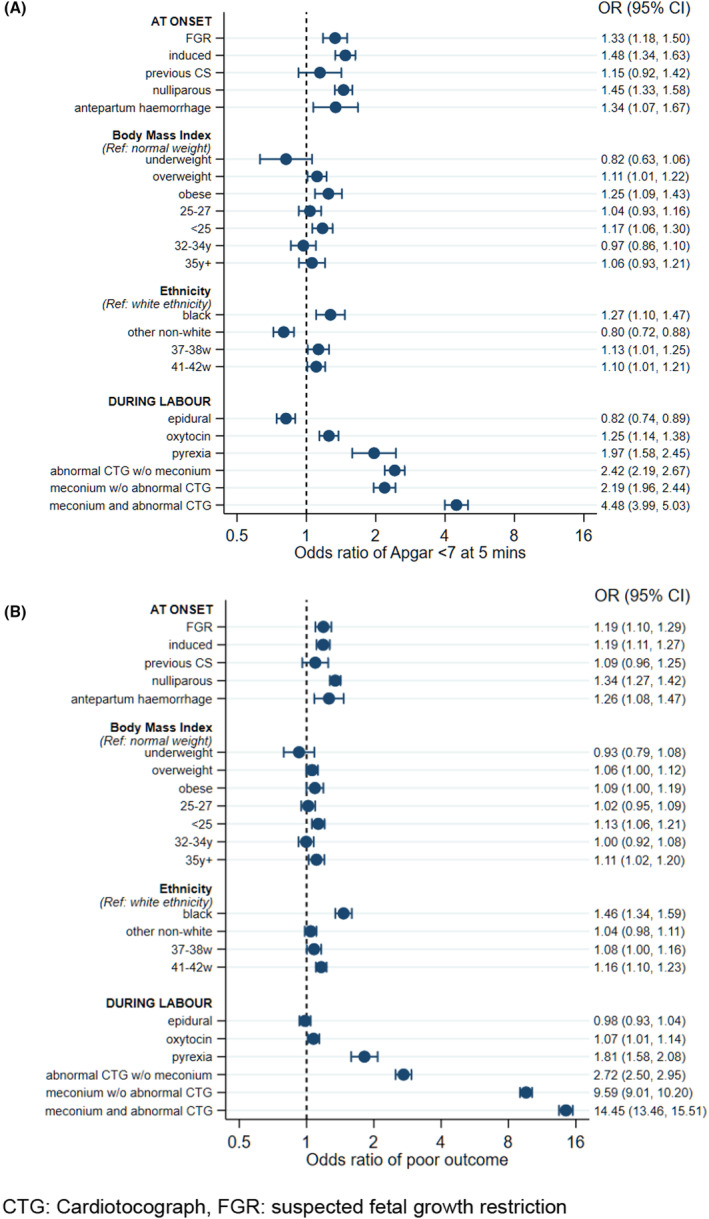
Adjusted odds ratios in women not undergoing emergency caesarean section (imputed data, *n* = 397 984) for (A) 5‐minute Apgar <7 and (B) composite poor outcome. Variable definitions are listed in Figure S2.

A total of 397 984 vaginal births were included in sensitivity analyses, which used multiple imputation for missing risk factors and outcomes (Figure 2). Results are broadly similar, though the association between antepartum haemorrhage and 5‐minute Apgar <7 was strengthened, (OR 1.34, 95% CI 1.07–1.67 versus OR 1.18, 95% CI 0.91–1.54 in the complete case analysis).

## 4 DISCUSSION

### 4.1 Main findings

This analysis of a large dataset shows that, in women giving birth vaginally, a range of risk factors is important in assessing fetal wellbeing during labour in addition to the fetal heart rate pattern. Maternal age <25 or more than 35 years, black ethnicity, nulliparity, suspected FGR, early‐term birth, induction of labour, oxytocin use, maternal pyrexia and meconium are independently associated with a higher risk of adverse outcomes. These risk factors have an association with adverse outcome comparable to an aFHRp alone. Our findings suggest that a strategy of assessing multiple risk factors could well improve detection and response to possible intrapartum fetal deterioration.

An association between poor neonatal outcome and meconium‐stained liquor on its own is well‐documented.^23^, ^24^, ^25^, ^26^ Our study found that meconium was associated with a 10‐fold increase in the composite adverse outcome. However, the interaction between this indicator and other risk factors is also important. Our data show that meconium occurring together with an aFHRp is associated with a 15‐fold increase in the composite outcome, suggesting that this combination might be considered sufficient grounds for expediting birth even in the absence of evidence for hypoxia (e.g. with a normal pH on fetal blood sampling). Certainly, the clinical significance of meconium‐stained amniotic fluid at 37 weeks is different from that at 42 weeks,^26^, ^27^, ^28^ when it is a common finding secondary to more advanced physiological maturity of the fetal bowel, and therefore the presence of meconium at 37 weeks is likely to be of greater clinical concern.^28^

A further important intrapartum risk factor identified by our study is maternal pyrexia in labour, which we found to be associated with an 80% higher odds of 5‐minute Apgar score <7 and a 90% higher odds of the composite poor outcome. This reinforces the need to treat potential infection in labour effectively (e.g. with timely intravenous antibiotics and resuscitative measures), while recognising that real‐time recognition of the causes of pyrexia (which may arise, for example, from chorioamnionitis or epidural anaesthesia) is not always straightforward.

In relation to antepartum risk factors, our findings may be useful in drawing attention to a possible area of investigation in relation to the ethnic patterning of poor outcomes. The association we identified between black ethnicity and adverse outcome (assessed using either 5‐minute Apgar score or the composite measure) adds to the concerning body of evidence of ethnic health inequalities by emphasising the heightened risks of pregnancy for non‐white women.^29^, ^30^, ^31^ Given that fetuses of women of black ethnicity are substantially more likely to pass meconium than those of white women,^27^ our study suggests that the mechanisms for this phenomenon, and the interventions which might be used to detect and address fetal risk in this key population, require more attention.

Our study adds to the evidence implicating FGR in perinatal morbidity and mortality.^32^, ^33^, ^34^ We found that suspected FGR (identified in our study by more than three antenatal ultrasound scans) is associated with a 35% higher odds of 5‐minute Apgar <7 and a 20% higher odds of the composite outcome. This is likely to arise because growth‐restricted babies have lower reserves to deal with intrapartum insults and therefore are more susceptible to hypoxia and subsequent poor outcomes. FGR (more specifically identified by growth trajectory and umbilical artery flow velocity abnormalities) is likely to be an even better predictor.

### 4.2 Strengths and limitations

Our study was based on a large dataset collected from all births in 17 (15) maternity units with a diverse population over 11 years in a single region of London, using agreed data definitions, validated in many previous studies. There was a low percentage of missing data in relation to key antenatal and intrapartum variables.

Our study has potential limitations. First, a small proportion of women had data missing for important risk factors such as ethnicity and BMI (which have been shown previously to have a significant effect on neonatal outcome).^35^, ^36^, ^37^ These cases were therefore excluded from the main analysis, but sensitivity analysis employing multiple imputation showed that our conclusions are likely valid for the total population studied. Secondly, the data are now dated, and changes in care over time might mean that some findings may not reflect current practice. Thirdly, data on death following the perinatal period and permanent brain injury were not available. The proxies chosen, while reasonable, remain surrogates for adverse outcomes. Fourthly, we studied only women with singleton, cephalic, term pregnancies, so the findings cannot be extrapolated to more complicated births. Further, as we excluded births involving emergency caesarean section, the results presented are unlikely to apply identically to these births. It is not possible to determine whether differences in outcomes arise because of the caesarean section intervention itself or because of differences in unmeasured confounders, including the circumstances that gave rise to the emergency. Finally, opportunities for patient and public involvement were limited by the nature of the available data, but it will clearly be vital to have full engagement with women and families in planning future research and improvement activities in response to these findings.

### 4.3 Interpretation

Our results demonstrate that labour outcomes are associated with specific antenatal and intrapartum risk factors even when the FHRp is taken into account. We have shown the importance of considering meconium alongside an aFHRp, and of accounting for other important risk factors, including an 80% higher risk of adverse outcomes with maternal pyrexia even allowing for fetal heart rate changes. These findings may indicate that a clinical prediction model might offer value in considering risk indicators in combination during labour to predict outcome. Important considerations in clinical prediction model development and evaluation include discrimination, calibration, validity and reliability. Use of the model in clinical practice would require repeated assessments of the risk factors as labour progresses, an approach that we have explored in our recent paper in this journal.^38^ The potential number of combinations of even the limited number of risk factors we have studied is huge. Artificial intelligence is likely to be of benefit in characterising these combinations and their interactions. Such a study is currently being planned and may, subject to appropriate data and analysis, yield a real‐time composite risk score. In the interim, we have provided clinically useful information on the indicators of risk and their relative importance.

## 5 CONCLUSION

Monitoring of the FHRp alone is not adequate to assess fetal wellbeing during labour in relation to the need to expedite birth. To optimise detection of deterioration, escalation, decision‐making and action, an aFHRp must be interpreted in the context of multiple risk factors including the presence of meconium, maternal pyrexia and suspected FGR, as well as important demographic and contextual risk factors.

## AUTHOR CONTRIBUTIONS

The study was conceived by PJS, MS, SJ, TD and MDW and planned by SJ, PJS, MS, TD, MDW, AW and LK. LK carried out the statistical analysis. The first draft was written by SJ and LK. All authors contributed to editing the drafts and approved the final article. All authors take responsibility for the final version.

## FUNDING INFORMATION

This study was supported by the Health Foundation grant to the University of Cambridge for The Healthcare Improvement Studies Institute. The Health Foundation is an independent charity committed to bringing about better health and healthcare for people in the UK. MDW is a National Institute for Health and Care Research Senior Investigator (NF‐SI‐0617‐10 026). This work was supported by core funding from the British Heart Foundation (RG/13/13/30194; RG/18/13/33946) and NIHR Cambridge Biomedical Research Centre (BRC‐1215‐20 014). The views expressed in this article are those of the authors and not necessarily those of the NHS, the National Institute for Health and Care Research or the Department of Health and Social Care. This work was also supported by Health Data Research UK, which is funded by the UK Medical Research Council, Engineering and Physical Sciences Research Council, Economic and Social Research Council, Department of Health and Social Care (England), Chief Scientist Office of the Scottish Government Health and Social Care Directorates, Health and Social Care Research and Development Division (Welsh Government), Public Health Agency (Northern Ireland), British Heart Foundation and Wellcome.

## CONFLICT OF INTEREST STATEMENT

PJS is the medical director and a shareholder in CaretekMedical, a company that has produced a smartphone app for women to assess their antenatal risk factors (http://www.caretekmedical.net/). Details of his additional interests can be found at https://obgyn.onlinelibrary.wiley.com/hub/journal/14710528/editors‐disclosures‐of‐interests#Philip_Steer. LK is funded by the NIHR BTRU in Donor Health and Behaviour (NIHR203337) and a BHF Chair award (CH/12/2/29428). This work was performed using resources provided by the Cambridge Service for Data Driven Discovery (CSD3) operated by the University of Cambridge Research Computing Service (www.csd3.cam.ac.uk), provided by Dell EMC and Intel using Tier‐2 funding from the Engineering and Physical Sciences Research Council (capital grant EP/P020259/1), and DiRAC funding from the Science and Technology Facilities Council (www.dirac.ac.uk). TJD was at the time of the work described in this paper the Vice‐President (clinical quality) of the Royal College of Obstetricians and Gynaecologists. SJ, MS, AW have no interests to declare.

## ETHICS APPROVAL

Governance Arrangements for Research Ethics Committees (GAfREC) were issued by the UK Health Departments in May 2011 and came into effect on 1 September 2011. This stated that ‘REC review is not required under the harmonised GAfREC for research limited to use of previously collected, non‐identifiable information.’ On 18 January 2012 we were informed by the Coordinator of the NRES Committee London that this exemption applied to the pseudo‐anonymised NW Thames dataset used in the current study, and that additional consent for each specific project using it was no longer required.

## Supporting information


Figure S1.

Figure S2.

Table 1.

Data S1.

Data S2.

Data S3.

Data S4.

Data S5.

Data S6.

Data S7.

## Data Availability

The pseudo‐anonymised data that support the study are available on request from the corresponding author. The original data are not publicly available in accordance with privacy and confidentiality restrictions.
