# Supplementary material for: Risk factors for a serious adverse outcome in neonates: a retrospective cohort study of vaginal births
Source: BJOG. 2023 May 8;130(12):1521–30. doi: 10.1111/1471-0528.17531 (PMC10952606; doi:10.1111/1471-0528.17531)
Supplement: Supplementary file 1 — Figure S1. [file BJO-130-1521-s005.docx]

**Supplementary Figure S1**: Flow chart summarising exclusions from analysis

585,291

Births recorded

472,771

Births meeting labour/birth criteria

428,742

Births eligible for analysis

302,137

Births included in complete case analysis

112,520 Exclusions not meeting criteria for labour/ birth:

67,910 No gestational age recorded (early miscarriage/ moved away)

1,489 Gestational age <24w (late miscarriage)

43,121 Births without labour (CS prior to labour onset)

44,029 Exclusions due to high-risk with different clinical management*:

29,022 Gestational age <37w (pre-term)

671 Gestational age >42w (post-term)

9,658 Multiple pregnancies

13,494 Breech/non-vertex presentation

104,098 Exclusions due to missing data:

8,251 Emergency CS

95,847 Non-emergency CS*

22,507 Emergency CS

324,644

Births eligible for analysis with non-missing data

* including 1,744 missing at least one component of outcome, 81,130 missing BMI, 13,505 missing other risk factors, Note breakdown exceeds total exclusions due to some labours having combinations of these.

** missing at least one outcome component; BMI: Body mass index, CS: Caesarean section
