# Supplementary material for: Risk factors for a serious adverse outcome in neonates: a retrospective cohort study of vaginal births
Source: BJOG. 2023 May 8;130(12):1521–30. doi: 10.1111/1471-0528.17531 (PMC10952606; doi:10.1111/1471-0528.17531)
Supplement: Supplementary file 2 — Figure S2. [file BJO-130-1521-s009.docx]

**Supplementary Figure S2: Definitions of risk factors**

**Risk factors at onset of labour**

- **Maternal age,** categorised into five groups: <25, 25-27, 28-31, 32-34, ≥35
- **Maternal ethnicity**, categorised as white, black, or other
- **Maternal booking body mass index**, categorised according to WHO classifications for underweight (<18.5), normal (18.5-24.9), overweight (25.0-29.9) and obese (≥30)
- **Parity,** dichotomised as nulliparous or not (parous)
- **Previous caesarean section**, dichotomised as any previous caesarean or none
- **Suspected fetal growth restriction (FGR)**. Preliminary analysis showed a strong correlation between the number of antenatal scans and the proportion of babies born weighing <5th percentile, thus enabling us to use >3 recorded antenatal scans as a surrogate risk factor for FGR.
- **Gestational age at delivery**, categorised as early term (37-38 weeks), mid-term (39-40 weeks), or late term (41-42 weeks)
- **Induction of labour,** dichotomised as spontaneous onset or induced
- **Antepartum haemorrhage** Intrapartum haemorrhage was included in the dataset with antenatal haemorrhage as ‘antepartum haemorrhage’ so this variable is not labour specific.

**Risk factors during labour**

- Epidural analgesia
- Oxytocin augmentation (which carries a risk of hyperstimulation)
- Maternal pyrexia (temperature ≥37.5^0^C)
- Abnormal fetal heart rate (FHR) as categorised at the time by the birth attendants
- Meconium stained amniotic fluid registered as significant by the birth attendants
