# Supplementary material for: Risk factors for a serious adverse outcome in neonates: a retrospective cohort study of vaginal births
Source: BJOG. 2023 May 8;130(12):1521–30. doi: 10.1111/1471-0528.17531 (PMC10952606; doi:10.1111/1471-0528.17531)
Supplement: Supplementary file 3 — Table 1. [file BJO-130-1521-s001.docx]

**Supplementary Table 1:** Summary of missing data in 397,984* labours leading to vaginal birth

|  | **Vaginal birth** |
| --- | --- |
| **Antenatal variables** |  |
| Suspected fetal growth restriction  No  Yes  Missing | 360,059 (90.5%)  33,203 (8.3%)  4,722 (1.2%) |
| Previous CS  No  Yes  Missing | 384,626 (96.6%)  13,299 (3.2%)  59 (<0.1%) |
| Nulliparity  No  Yes  Missing | 227,903 (57.3%)  170,022 (42.7%)  59 (<0.1%) |
| Antepartum haemorrhage  No  Yes  Missing | 387,452 (97.4%)  8,054 (2.0%)  2,478 (0.6%) |
| Booking body mass index  Normal (18-24kg/m^2^)  Underweight (<18kg/m^2^)  Overweight (25-29kg/m^2^)  Obese (≥30kg/m^2^)  Missing | 201,382 (50.6%)  10,658 (2.7%)  76,381 (19.2%)  28,433 (7.1%)  81,130 (20.4%) |
| Maternal age  <25  25-27  28-31  32-34  ≥35  Missing | 95,502 (24.0%)  76,077 (19.1%)  114,558 (28.8%)  61,509 (15.5%)  50,333 (12.7%)  5 (<0.1%) |
| Maternal ethnicity  White  Black  Other  Missing | 283,808 (71.3%)  22,135 (5.6%)  80,849 (20.3%)  11,192 (2.8%) |
| Gestational age at delivery  37-38w  39-40w  41-42w  Missing | 70,323 (17.7%)  230,518 (57.9%)  95,537 (24.1%)  1,606 (0.4%) |
| **Intrapartum variables** |  |
| Induction of labour  No  Yes  Missing | 328,061 (82.4%)  69,795 (17.5%)  128 (<0.1%) |
| Epidural use  No  Yes  Missing | 310,703 (78.1%)  87,281 (21.9%)  0 (0%) |
| Oxytocin use  No  Yes  Missing | 312,260 (78.5%)  84,992 (21.4%)  732 (0.2%) |
| Maternal pyrexia  No  Yes  Missing | 393,443 (98.9%)  4,195 (1.1%)  346 (0.1%) |
| Abnormal FHR  No  Yes  Missing | 329,138 (82.7%)  68,558 (17,2%)  288 (0.1%) |
| Meconium-stained amniotic fluid  No  Yes  Missing | 332,608 (83.6%)  65,136 (16.4%)  240 (0.1%) |

CS: Caesarean section, FHR: Fetal heart rate

* 302,137 complete cases + 95,847 with at least one missing value.
